# Immune and Redox Responses of *Galleria mellonella* to Caffeic Acid: Concentration- and Time-Dependent Patterns

**DOI:** 10.3390/biomedicines14092116

**Published:** 2026-09-19

**Authors:** Fatih Battal, Serhat Kaya

**Affiliations:** 1 Department of Pediatrics, Faculty of Medicine, Çanakkale Onsekiz Mart University, Çanakkale 17020, Türkiye; 2 Department of Biology, Faculty of Science, Çanakkale Onsekiz Mart University, Çanakkale 17400, Türkiye; serhatkaya@comu.edu.tr

**Keywords:** *Galleria mellonella*, caffeic acid, innate immunity, hemocytes, encapsulation, melanization, oxidative stress, catalase, superoxide dismutase, lipid peroxidation

## Abstract

**Background/Objectives:** Caffeic acid is a naturally occurring phenolic compound with antioxidant and immunomodulatory properties, but its integrated effects on innate immune and redox-related physiology in *Galleria mellonella* remain unclear. This study evaluated concentration- and time-related effects of caffeic acid on immune and biochemical endpoints. **Methods:** Last-instar larvae were exposed to 0.1–5 mg/mL caffeic acid, with 20% ethanol as the vehicle comparator. Total hemocyte count (THC), Sephadex A-25 bead encapsulation, and melanization were assessed as immune endpoints; bead responses were evaluated at 4 and 24 h using independent cohorts. Total protein, superoxide dismutase (SOD), catalase (CAT), and malondialdehyde (MDA) were measured at 24 h. Experimental run was included as a fixed blocking factor in the primary inferential models; THC and biochemical endpoints were analyzed using endpoint-appropriate run-adjusted linear or rank-based models, whereas encapsulation and melanization were analyzed using ordinal and binomial generalized estimating equations (GEEs), respectively; *n* = 16 larvae per treatment for each endpoint. **Results:** THC increased significantly only at 0.5 mg/mL relative to the vehicle. Encapsulation showed a significant treatment × time interaction; all concentrations shifted encapsulation toward higher categories at 4 h, whereas 2.5 and 5 mg/mL shifted toward lower categories at 24 h. Melanization showed no significant treatment × time interaction; only 2.5 mg/mL increased melanization odds at 4 h, with no treatment–vehicle differences at 24 h. CAT increased at 2.5 and 5 mg/mL, and SOD at 2.5 mg/mL. MDA and total protein showed overall heterogeneity without significant individual treatment–vehicle differences. **Conclusions:** Caffeic acid produced non-uniform, endpoint-specific modulation of immune and antioxidant responses, with temporal dependence most evident in encapsulation.

## 1. Introduction

Caffeic acid (3,4-dihydroxycinnamic acid) is a naturally occurring hydroxycinnamic acid widely distributed in plant-derived foods, beverages, and natural products, including coffee, fruits, vegetables, wine, olive oil, and propolis. It has attracted considerable scientific interest because of its antioxidant, anti-inflammatory, antimicrobial, and immunomodulatory activities [1,2,3,4]. Many of these properties are associated with its phenolic structure, particularly the catechol moiety, which enables interactions with reactive oxygen species (ROS) and other redox-active molecules [1,3,4].

Reactive oxygen species are generated during normal cellular metabolism but can increase substantially under biological or environmental stress. At controlled levels, ROS participate in cellular signaling and host defense, whereas excessive production can damage proteins, membrane lipids, and nucleic acids. Antioxidant homeostasis therefore depends on coordinated enzymatic and non-enzymatic defenses. Among the principal antioxidant enzymes, superoxide dismutase (SOD) converts superoxide to hydrogen peroxide, whereas catalase (CAT) contributes to subsequent hydrogen peroxide removal. Malondialdehyde (MDA), a product of lipid peroxidation, is commonly used as an indirect indicator of oxidative damage to membrane lipids [5,6,7,8].

In insects, redox regulation is closely integrated with innate immunity. Reactive oxygen species can contribute to antimicrobial defense and immune signaling, while cellular immune activation and melanization can themselves alter the local redox environment. This relationship is particularly relevant during encapsulation and activation of the prophenoloxidase system, where cellular defense and redox-active biochemical reactions interact [9,10]. Consequently, simultaneous assessment of immune-associated and oxidative endpoints can provide a more informative view of the physiological effects of bioactive compounds than evaluation of either system in isolation.

The greater wax moth (*Galleria mellonella*) has become a widely used in vivo model for studies of innate immunity, host–pathogen interactions, toxicology, and bioactive compounds. Its cellular immune response is mediated primarily by hemocytes, which participate in phagocytosis, nodulation, and encapsulation. Humoral and biochemical defenses include antimicrobial peptides, coagulation, and the prophenoloxidase system, while melanization is functionally integrated with cellular responses to non-self material [11,12,13,14]. Accordingly, total hemocyte count (THC) provides information on circulating cellular immune status, encapsulation provides a functional measure of hemocyte-mediated defense against large foreign targets, and melanization represents an additional functional outcome associated with activation of the prophenoloxidase system [10,12,15].

Studies in *G. mellonella* have demonstrated that infectious, chemical, and dietary challenges can alter hemocyte responses, antioxidant enzyme activities, and lipid peroxidation, indicating that immune and redox physiology can respond simultaneously to environmental or pharmacological perturbation [8,16,17,18,19]. This integration is particularly relevant for phenolic compounds because their biological effects may extend beyond direct radical scavenging to include concentration-dependent modulation of cellular and endogenous redox responses.

Although the antioxidant properties of caffeic acid have been extensively investigated in chemical, cellular, and mammalian systems, considerably less is known about its effects on the integrated immune and redox physiology of *G. mellonella* [1,3,4,20]. Moreover, antioxidant activity demonstrated in chemical systems does not necessarily predict the physiological response of an intact organism, and changes in individual immune or antioxidant parameters cannot alone establish generalized immunostimulatory or antioxidant effects. To our knowledge, the concentration-dependent effects of free caffeic acid on hemocyte abundance, encapsulation, melanization, antioxidant enzyme activities, lipid peroxidation, and total hemolymph protein have not previously been evaluated together in *G. mellonella*.

Accordingly, the present study investigated the concentration-dependent effects of caffeic acid on immune-associated and redox-related physiology in *G. mellonella*. Total hemocyte count, encapsulation, and melanization were evaluated as cellular and functional immune endpoints, together with total hemolymph protein, SOD and CAT activities, and MDA as biochemical and redox-related parameters. We hypothesized that caffeic acid would produce concentration-dependent changes in these endpoints and that the magnitude and direction of the responses would differ among physiological parameters.

## 2. Materials and Methods

### 2.1. Insect Rearing and Experimental Model

A laboratory colony of *G. mellonella* maintained at the Insect Physiology Laboratory, Department of Biology, Faculty of Science, Çanakkale Onsekiz Mart University, Türkiye, was used throughout the study. Adult moths emerging from pupae were maintained in 1 L glass jars at a ratio of four females to two males, together with approximately 2 g of darkened honeycomb. After larval emergence, the insects were provided with an artificial diet consisting of 250 g natural darkened honeycomb, 1000 g wheat bran, 300 mL honey, 300 mL distilled water, and 300 mL glycerol.

The colony was maintained at 30 ± 1 °C, approximately 65% relative humidity, and in complete darkness. Under these conditions, larvae reached the last larval instar in approximately 3 weeks. Last-instar larvae were identified using body size and head-capsule morphometric characteristics in accordance with published descriptions of stage-specific changes in body and head-capsule dimensions in *G. mellonella* [21]. Healthy larvae weighing 0.18 ± 0.02 g and showing no visible melanization, physical injury, or other abnormal appearance were selected for the experiments.

Eligible larvae were randomly allocated to experimental groups before treatment. All microscopic evaluations of encapsulation and melanization were performed by the same investigator. Because the investigator performed the experimental treatments and subsequent microscopic assessments, these evaluations were not blinded to treatment allocation.

### 2.2. Caffeic Acid Preparation and Experimental Design

Caffeic acid (catalogue no. C0625, purity ≥98.0% by HPLC; Sigma-Aldrich, Taufkirchen, Germany) was used throughout the study. Experimental solutions were prepared at concentrations of 0.1, 0.25, 0.5, 1, 2.5, and 5 mg/mL. The highest concentration (5 mg/mL) was prepared in 20% ethanol made with sterile distilled water, and the lower experimental concentrations were subsequently prepared using the same 20% ethanol vehicle.

Caffeic acid solutions were freshly prepared immediately before injection in each independent experimental run and were protected from light until administration. The solutions were not filter-sterilized, and their pH was not measured. Caffeic acid dissolved completely in the vehicle under the preparation conditions used, with no visible precipitation observed at any concentration, including 5 mg/mL. Chemical stability during the short preparation-to-administration interval was not independently assessed. The concentration range was established empirically. A concentration of 5 mg/mL was selected as the highest concentration that could be completely dissolved under the preparation conditions and that did not produce visible acute adverse effects in preliminary observations. The lower experimental concentrations were subsequently generated by serial dilution of this highest concentration in the same 20% ethanol vehicle.

A 20% ethanol group served as the vehicle control, whereas untreated larvae served as the negative control. Because all caffeic acid-treated groups received the same ethanol vehicle, the ethanol vehicle was defined a priori as the primary comparator for evaluating caffeic-acid-associated effects.

Overall, 512 larvae were used across the four independent experimental cohorts: 128 for THC, 128 for the 4 h bead assay, 128 for the 24 h bead assay, and 128 for the biochemical analyses.

#### 2.2.1. Immune-Response Experiments

Prior to injection, larvae were surface-disinfected with 70% ethanol and briefly immobilized on an ice pack. Under a stereomicroscope (Leica EZ4, Leica Microsystems, Wetzlar, Germany), 5 μL of the corresponding experimental solution was injected into the terminal abdominal proleg using a 25-μL Hamilton microsyringe (Hamilton Company, Reno, NV, USA).

The administered amounts of caffeic acid were 0.5, 1.25, 2.5, 5, 12.5, and 25 µg per larva for the 0.1, 0.25, 0.5, 1, 2.5, and 5 mg/mL groups, respectively. Based on the mean larval body mass of 0.18 g, these corresponded to approximate body-mass-normalized doses of 2.8, 6.9, 13.9, 27.8, 69.4, and 138.9 µg/g body mass, respectively.

Four independent experimental runs were conducted on different days, with approximately 1 week between successive runs. Each run included four larvae from each experimental group. Thus, each treatment group comprised 16 individual larvae distributed across four experimental runs. Within each run, the four larvae belonging to a given treatment were maintained together in a Petri dish under the same controlled environmental conditions following treatment.

For immune-response analyses, separate larval cohorts were used for total hemocyte count and for the bead-based encapsulation/melanization assays. For the bead assays, independent cohorts were used for the 4 and 24 h assessments because larvae were sacrificed during bead recovery.

#### 2.2.2. Hemolymph Collection and Sample Preparation for Biochemical Analyses

For biochemical analyses, hemolymph was collected 24 h after treatment. Each larva was processed individually, and samples from different larvae were not pooled. Hemolymph was obtained by puncturing the mid-body region with a sterile needle, and approximately 30 μL was transferred into an individual sterile microcentrifuge tube containing 0.001 mg powdered N-phenylthiourea (PTU) to suppress melanization (PTU; approximately 0.22 mM based on a 30 µL hemolymph volume). PTU was weighed separately for each tube using a Sartorius analytical balance and was added directly in powdered form without preparation of a stock solution.

Samples were centrifuged at 10,000× *g* for 5 min, after which the resulting supernatants were collected and stored at −20 °C until analysis. Individual larval supernatants were used for determination of total protein concentration, superoxide dismutase (SOD) activity, catalase (CAT) activity, and malondialdehyde (MDA) levels.

Biochemical analyses were likewise performed using 16 individual larvae per treatment, distributed across four independent experimental runs with four larvae per treatment in each run. Each larva was treated and processed as an independent biological replicate, and the same individual hemolymph supernatant was used for the total protein, SOD, CAT, and MDA measurements.

### 2.3. Total Hemocyte Count

Total hemocyte count (THC) was determined using a Neubauer hemocytometer (Marienfeld Superior, Lauda-Königshofen, Germany). Hemolymph was collected from the anterior abdominal proleg by puncturing each larva with a sterile needle. A 4 μL aliquot of hemolymph was immediately transferred into a microcentrifuge tube containing 36 μL of anticoagulant solution consisting of 0.098 M NaOH, 0.186 M NaCl, 0.017 M Na_2_EDTA, and 0.041 M citric acid (pH 4.5), corresponding to a 1:10 dilution.

The suspension was gently mixed by pipetting to minimize cell aggregation, and 10 μL was loaded into a Neubauer hemocytometer. Hemocytes were counted under a phase-contrast microscope (SOPTOP CX-40, Ningbo Sunny Instruments Co., Ltd., Yuyao, China). For each larva, a single hemocytometer count was performed, and all 25 counting squares of the central grid were examined. The total hemocyte concentration was calculated according to the chamber volume and dilution factor and expressed as ×10^5^ cells/mL. Sixteen individual larvae were evaluated per treatment across four independent experimental runs, with four larvae analyzed per run.

### 2.4. Encapsulation Assay

Encapsulation responses were assessed using Sephadex A-25 chromatography beads (40–120 μm; Sigma-Aldrich, St. Louis, MO, USA) as standardized non-self targets. Before use, Sephadex A-25 beads were stained for 1 h in 1% (*w*/*v*) Coomassie Brilliant Blue G-250 prepared in PBS. Following staining, the beads were washed repeatedly with PBS until no visible excess dye remained in the wash solution. The stained beads were then stored at 4 °C until use. This staining procedure was used to facilitate microscopic visualization of the cellular layers surrounding the beads.

Approximately 15–20 stained Sephadex A-25 beads suspended in 10 μL phosphate-buffered saline (PBS, pH 7.4) were injected into the terminal abdominal proleg. Because the number of beads aspirated into the syringe was not counted individually before each injection, the administered bead number represents an approximate range rather than an exact per-larva dose.

Independent cohorts of larvae were used for the 4 and 24 h evaluations. At the designated assessment time, larvae were sacrificed and dissected by a dorsal longitudinal incision, and recovered beads were collected for microscopic examination. Consequently, observations at 4 and 24 h were obtained from different larvae and do not represent longitudinal measurements of the same individuals.

Each recovered bead was examined individually under a phase-contrast microscope. The hemocyte layers surrounding each bead were counted before assignment to an encapsulation category. Beads showing no capsule or only a few adherent hemocytes were classified as unencapsulated. Capsules consisting of 2–8 cellular layers were classified as weakly encapsulated, whereas capsules consisting of ≥9 cellular layers were classified as strongly encapsulated. Encapsulation was scored when the cellular capsule covered at least 50% of the bead circumference.

All microscopic assessments were performed by a single investigator who was aware of treatment allocation. Sixteen larvae were evaluated per treatment at each assessment time.

### 2.5. Melanization Assay

Melanization was evaluated concurrently with encapsulation using the Sephadex A-25 beads recovered from the same individual larvae. Thus, within each larva and assessment time, encapsulation and melanization were evaluated on the same recovered foreign targets, providing paired assessments of these two defense responses.

Each recovered bead was examined microscopically for visible melanization. Beads exhibiting visible darkening associated with the surrounding hemocyte capsule were classified as melanized, whereas beads without visible darkening were classified as non-melanized. Melanization was therefore recorded as a binary bead-level outcome.

As in the encapsulation assay, independent larval cohorts were used for the 4 and 24 h assessments. All evaluations were performed by the same investigator, who was aware of treatment allocation.

### 2.6. Biochemical Analyses

#### 2.6.1. Determination of Total Protein Concentration

Total hemolymph protein concentration was determined using the Bradford method [22]. For each individual sample, 5 μL of supernatant was transferred into a 96-well microplate, followed by 155 μL distilled water and 40 μL Bradford reagent. The plate was incubated for 20 min at room temperature, after which absorbance was measured at 595 nm.

A calibration curve was generated using bovine serum albumin (BSA) standards ranging from 0.1 to 1.6 mg/mL, prepared from a 2 mg/mL BSA stock solution. BSA concentration was used as the independent variable and absorbance at 595 nm as the dependent variable. Protein concentration was calculated as [protein, mg/mL] = (A595 − 0.027)/0.4327 (R^2^ = 0.97). For example, for a representative sample with an A595 value of 0.4512, the protein concentration was calculated as (0.4512 − 0.027)/0.4327 = 0.9804 mg/mL.

#### 2.6.2. Superoxide Dismutase Activity

Superoxide dismutase activity was determined using a xanthine–xanthine oxidase/NBT assay based on the method of Flöhe and Ötting [23]. A 0.3 mM xanthine working solution was freshly prepared from a 3 mM stock solution. Additional assay solutions included 0.6 mM Na_2_EDTA, 400 mM Na_2_CO_3_, 1 mg/mL BSA, 0.15 mM nitro blue tetrazolium (NBT), and 0.8 mM CuCl_2_. The SOD reagent was freshly prepared on the day of each experiment by combining 10 mL 0.3 mM xanthine, 5 mL 0.6 mM Na_2_EDTA, 3 mL 400 mM Na_2_CO_3_, 1.5 mL 1 mg/mL BSA, and 5 mL 0.15 mM NBT. These concentrations and reagent proportions are specified in the laboratory SOP.

Xanthine oxidase solution was prepared from a 40-U enzyme preparation by diluting 32 μL to a final volume of 1 mL with cold distilled water. For each assay, 6.5 μL sample supernatant was added to a microplate well, followed by 190 μL SOD reagent and 3.5 μL xanthine oxidase solution. The reaction was incubated for 20 min at room temperature under light, after which 6.5 μL of 0.8 mM CuCl_2_ was added to terminate the reaction. Absorbance was measured at 560 nm using a Multiskan GO UV/Vis microplate spectrophotometer (Thermo Scientific, Finland). SOD activity was calculated using the protocol-defined conversion coefficient according to

SOD activity = (A_560_ × 26.6)/total protein concentration

and expressed as U/mg protein.

#### 2.6.3. Catalase Activity

Catalase activity was determined using a kinetic hydrogen peroxide decomposition assay based on the method of Aebi [24]. A 10 mM phosphate buffer was prepared using Na_2_HPO_4_ and NaH_2_PO_4_. The dibasic solution was prepared by dissolving 138 mg Na_2_HPO_4_ in 100 mL distilled water, and the monobasic solution by dissolving 142 mg NaH_2_PO_4_ in 100 mL distilled water. The monobasic solution was added gradually to the dibasic solution until pH 7.0 was reached.

A 30 mM H_2_O_2_ solution was freshly prepared for each experiment. The laboratory SOP confirms both the 30 mM H_2_O_2_ preparation and the assay volumes. For each sample, 6.5 μL supernatant and 60 μL phosphate buffer were added to a microplate well. Immediately before kinetic measurement, 133.5 μL of 30 mM H_2_O_2_ was added.

Absorbance was monitored kinetically at 242 nm over a 2 min measurement period using a Multiskan GO UV/Vis microplate spectrophotometer (Thermo Fisher Scientific Oy, Vantaa, Finland) controlled by SkanIt™ Software for Microplate Readers, version 8.0. For each reading, absorbance was normalized to the corresponding total protein concentration according to

Normalized CAT value = (A_242_ × 0.0394)/total protein concentration.

CAT activity was subsequently calculated as

CAT activity = (maximum normalized value − minimum normalized value)/2

where 2 represents the 2 min kinetic measurement period. CAT activity was expressed as assay-defined units normalized to total protein (U/mg protein). One unit represents the activity calculated from the change in A242 over the 2 min kinetic measurement using the protocol-specific conversion coefficient of 0.0394, normalized to the corresponding total protein concentration.

#### 2.6.4. Determination of Malondialdehyde (MDA) Levels

Malondialdehyde levels were determined using the thiobarbituric acid (TBA) method based on Buege and Aust [25]. The TBA–TCA reagent was freshly prepared on the day of each experiment. Briefly, 7.5 g trichloroacetic acid (TCA) was dissolved and combined with 0.187 g TBA, after which the final volume was adjusted to 50 mL with 0.25 N HCl. The resulting reagent contained approximately 15% (*w*/*v*) TCA and 0.374% (*w*/*v*) TBA. These preparation conditions correspond to the laboratory SOP.

For each individual sample, 75 μL supernatant was mixed with 150 μL TBA–TCA reagent and incubated at 90 °C for 20 min. After heating, samples were allowed to cool to room temperature before absorbance measurement. No additional post-heating centrifugation or assay-specific turbidity correction was performed. Absorbance was subsequently measured at 532 nm using a Multiskan GO UV/Vis microplate spectrophotometer (Thermo Fisher Scientific Oy, Vantaa, Finland). No assay-specific blank or caffeic-acid spike interference control was included.

MDA levels were calculated according to

MDA = (A_532_ × 1.56)/total protein concentration

and expressed as nmol/mg protein/mL.

### 2.7. Statistical Analysis

Statistical analyses were performed using Python version 3.13.5, with SciPy version 1.17.0 and statsmodels version 0.14.6.. The individual larva was considered the biological unit of observation, while multiple beads recovered from the same larva were treated as clustered subsamples. The ethanol vehicle was defined a priori as the primary comparator. Experimental run was included as a fixed blocking factor in all primary inferential models; because only four run levels were available, run was not modeled as a random-effect variance component. Planned comparisons against the vehicle were adjusted using the Holm procedure, and statistical significance was accepted at *p* < 0.05.

THC was analyzed using a linear model including treatment and experimental run as fixed effects after Shapiro–Wilk and Levene tests confirmed acceptable assumptions. The overall treatment effect was summarized using omega-squared (ω^2^).

Encapsulation was analyzed as an ordered bead-level outcome (unencapsulated < weakly encapsulated < strongly encapsulated) using a cumulative-logit generalized estimating equation (GEE) with larva as the clustering variable, robust covariance estimation, and treatment, assessment time, treatment × time, and experimental run as fixed model terms. Planned vehicle contrasts were performed within each assessment time and are reported as odds ratios (ORs) with 95% confidence intervals (CIs). A larva-level Encapsulation Severity Index (ESI) was additionally used as a complementary descriptive analysis.

Melanization was analyzed as a binary bead-level outcome using a binomial GEE with an exchangeable working correlation structure, larva as the clustering variable, robust covariance estimation, and treatment, assessment time, treatment × time, and experimental run as fixed model terms. Planned vehicle contrasts were evaluated within each assessment time, and comparisons between the independent 4 and 24 h cohorts were also performed.

Total protein and SOD were analyzed using HC3-robust linear models including treatment and experimental run as fixed effects. CAT activity was summarized non-parametrically using the Kruskal–Wallis test with epsilon-squared (ε^2^H) and was additionally analyzed using a rank-based HC3-robust fixed-block model to account for experimental run. MDA was analyzed using a linear model including treatment and experimental run as fixed effects because its distributional assumptions were acceptable. For these endpoints, planned vehicle contrasts were obtained from the corresponding run-adjusted models and adjusted using the Holm procedure.

Continuous data are presented as mean ± SD where appropriate, whereas CAT is additionally summarized using median and interquartile range. Effect sizes and 95% CIs are reported for the principal treatment effects and planned contrasts. Complete statistical outputs are provided in Supplementary Files S1–S4 (Tables S1–S19).

## 3. Results

### 3.1. Effect of Caffeic Acid on Total Hemocyte Count

Total hemocyte count (THC) data showed no significant deviation from normality in any experimental group (Shapiro–Wilk tests, all *p* > 0.05), and the assumption of homogeneity of variances was satisfied (Levene’s test: F(7, 120) = 0.85, *p* = 0.552). Descriptive statistics, assumption checks, run-adjusted model results, planned comparisons against the ethanol vehicle, and the complete larva-level analysis dataset are provided in Supplementary File S4 (Tables S16–S19 and Analysis Dataset).

Accordingly, THC was analyzed using a linear model including treatment and experimental run as fixed effects, with run serving as a blocking factor. After adjustment for experimental run, treatment had a significant effect on THC (F(7, 117) = 9.66, *p* < 0.001, ω^2^ = 0.324), whereas the run effect was not significant (F(3, 117) = 0.50, *p* = 0.684; Supplementary File S4, Table S18).

Because caffeic acid was administered in 20% ethanol, the ethanol vehicle was used as the primary comparator. Planned comparisons against the ethanol vehicle, with Holm adjustment for multiple testing, showed that THC was significantly higher at 0.5 mg/mL (370.0 ± 33.0 × 10^5^ cells/mL) than in the ethanol vehicle group (290.4 ± 30.3 × 10^5^ cells/mL; mean difference = 79.6 × 10^5^ cells/mL, 95% CI 53.3–105.8 × 10^5^ cells/mL, Holm-adjusted *p* < 0.001; Supplementary File S4, Table S19). None of the other caffeic acid concentrations differed significantly from the ethanol vehicle after adjustment (all Holm-adjusted *p* > 0.05). The untreated control likewise did not differ significantly from the ethanol vehicle. 

**Figure 1:**
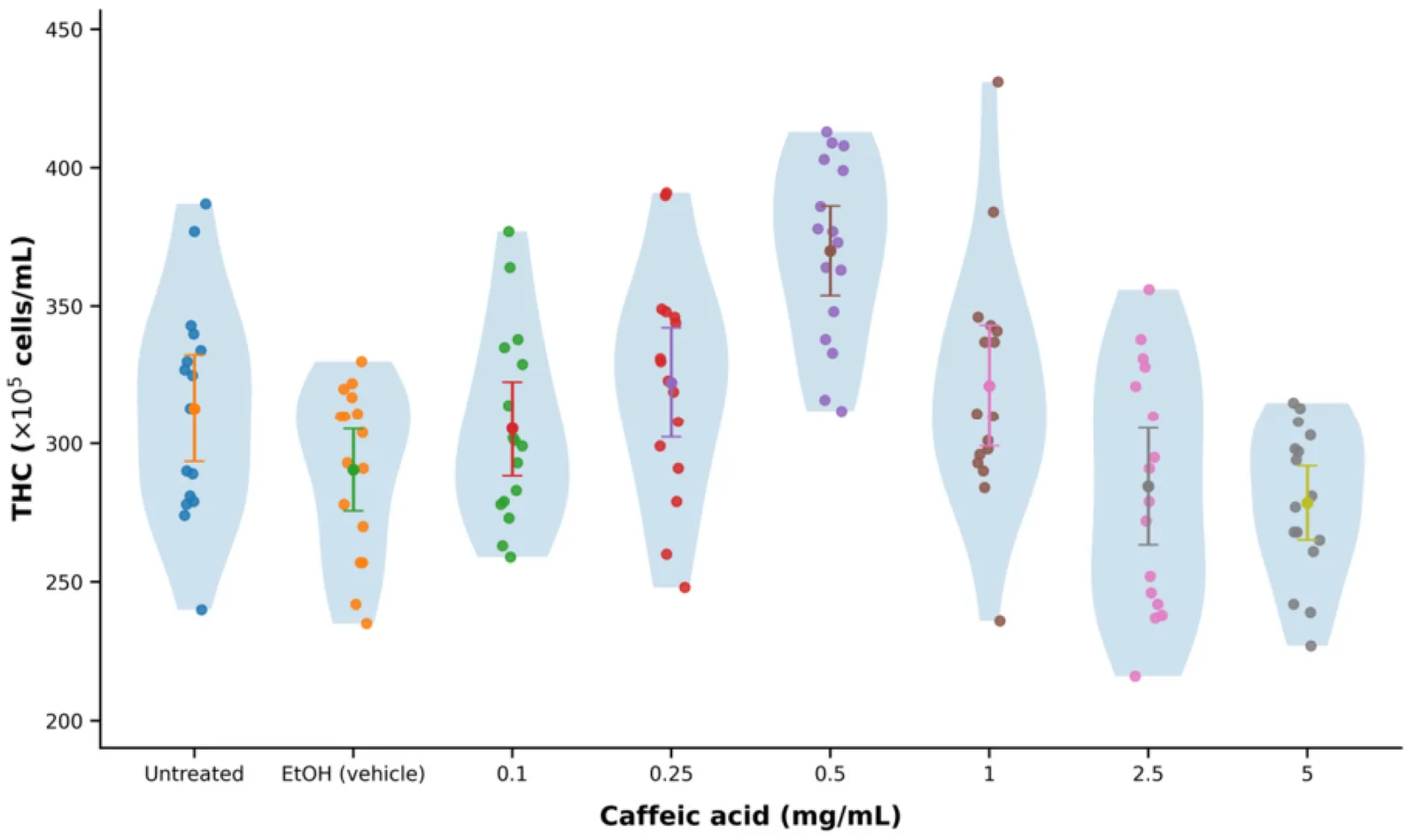
Effect of caffeic acid on total hemocyte count (THC) in *G. mellonella* larvae 24 h after treatment. Data are presented as mean ± SD (*n* = 16 larvae per group). THC was analyzed using a linear model including treatment and experimental run as fixed effects, with run serving as a blocking factor. The treatment effect was significant after adjustment for experimental run (F(7, 117) = 9.66, *p* < 0.001, ω^2^ = 0.324), whereas the run effect was not significant (F(3, 117) = 0.50, *p* = 0.684). Planned comparisons against the ethanol vehicle were adjusted using the Holm procedure. THC was significantly higher only at 0.5 mg/mL caffeic acid than in the ethanol vehicle group (adjusted mean difference = 79.6 × 10^5^ cells/mL, 95% CI 53.3–105.8 × 10^5^ cells/mL, Holm-adjusted *p* < 0.001). Detailed statistical results are provided in Supplementary File S4 (Tables S16–S19).

Overall, the THC response was non-monotonic, with the highest mean hemocyte count observed at 0.5 mg/mL rather than progressively increasing with caffeic acid concentration (Figure 1).

### 3.2. Effect of Caffeic Acid on Encapsulation Response

Encapsulation responses showed marked treatment- and time-dependent variation. Because encapsulation was recorded as an ordered categorical outcome (unencapsulated < weak < strong) for multiple beads recovered from the same larva, the primary analysis was performed using a cumulative-logit generalized estimating equation (GEE) with larva as the clustering unit and robust covariance estimation. The model revealed a significant treatment × time interaction (Wald χ^2^_7_ = 212.68, *p* < 0.001), indicating that the association between caffeic acid treatment and encapsulation category differed between the 4 and 24 h cohorts (Supplementary File S1, Table S7). The treatment-specific odds ratios relative to the ethanol vehicle are summarized in Table 1. Detailed descriptive statistics, complementary analyses, planned contrasts, and full ordinal GEE outputs for encapsulation are provided in Supplementary File S1 (Tables S1–S7), with the primary ordinal GEE results presented in Table S7.

At 4 h, all caffeic acid concentrations significantly increased the odds of a bead being assigned to a higher encapsulation category relative to the ethanol vehicle (Table 1). The odds ratios were 2.21 (95% CI 1.75–2.80) at 0.1 mg/mL, 3.17 (95% CI 2.48–4.06) at 0.25 mg/mL, 3.19 (95% CI 2.53–4.02) at 0.5 mg/mL, 2.16 (95% CI 1.76–2.64) at 1 mg/mL, 1.90 (95% CI 1.52–2.38) at 2.5 mg/mL, and 3.04 (95% CI 2.38–3.88) at 5 mg/mL; all Holm-adjusted *p* values were < 0.001. Thus, caffeic acid treatment was associated with a shift toward stronger encapsulation categories relative to the vehicle during the 4 h response.

At 24 h, the direction of the response differed according to concentration (Table 1). The odds of assignment to a higher encapsulation category remained significantly greater than the ethanol vehicle at 0.1 mg/mL (OR = 1.73, 95% CI 1.34–2.25), 0.25 mg/mL (OR = 2.17, 95% CI 1.62–2.92), 0.5 mg/mL (OR = 1.71, 95% CI 1.27–2.29), and 1 mg/mL (OR = 1.70, 95% CI 1.25–2.29). In contrast, the odds were significantly lower than the vehicle at 2.5 mg/mL (OR = 0.59, 95% CI 0.44–0.78) and 5 mg/mL (OR = 0.37, 95% CI 0.27–0.49); all Holm-adjusted *p* values were ≤0.001. These findings indicate that the 24 h response was characterized by maintenance of comparatively stronger encapsulation at low-to-intermediate caffeic acid concentrations, but a shift toward lower encapsulation categories at the two highest concentrations.

The untreated control also showed greater odds of assignment to a higher encapsulation category than the ethanol vehicle at both 4 h (OR = 2.14, 95% CI 1.66–2.76, Holm-adjusted *p* < 0.001) and 24 h (OR = 1.77, 95% CI 1.25–2.49, Holm-adjusted *p* = 0.001), indicating that the vehicle itself influenced the encapsulation response.

As a complementary larva-level summary, the Encapsulation Severity Index (ESI) also showed significant effects of treatment, time, and their interaction (HC3-robust treatment: F_7_,_240_ = 33.82, *p* < 0.001; time: F_1_,_240_ = 117.86, *p* < 0.001; treatment × time: F_7_,_240_ = 28.18, *p* < 0.001) (Supplementary File S1, Tables S1 and S2). The ESI values and the corresponding distributions of unencapsulated, weakly encapsulated, and strongly encapsulated beads are presented in Table 2. At 24 h, the reduction in encapsulation severity at 2.5 and 5 mg/mL was mainly attributable to redistribution from strongly toward weakly encapsulated beads rather than to a generalized increase in unencapsulated beads.

### 3.3. Effect of Caffeic Acid on Melanization

Melanization remained high across all experimental groups at both assessment times, with mean larval melanization percentages ranging from 94.25% to 99.49% at 4 h and from 93.11% to 96.50% at 24 h (Table 3). Individual larva-level counts of non-melanized and melanized beads, together with the corresponding derived percentages and full statistical outputs, are provided in Supplementary File S2 (Tables S8–S11), with the primary binomial GEE results presented in Table S11. Because melanization was recorded as a binary bead-level outcome (melanized vs. non-melanized), the data were analyzed using a binomial generalized estimating equation (GEE) with larva as the clustering unit. The overall treatment × time interaction was not statistically significant (Wald χ^2^_7_ = 11.26, *p* = 0.128), indicating that there was no sufficient evidence for an overall difference in the treatment-associated pattern of melanization between the independently sampled 4 and 24 h cohorts.

At 4 h, planned contrasts against the ethanol vehicle showed that melanization odds were significantly higher only at 2.5 mg/mL (OR = 8.35, 95% CI 2.17–32.17, Holm-adjusted *p* = 0.014). Although the estimated odds were also higher at 5 mg/mL (OR = 3.55, 95% CI 1.33–9.50) and 1 mg/mL (OR = 3.33, 95% CI 1.21–9.12), these differences did not remain statistically significant after Holm adjustment (adjusted *p* = 0.070 and 0.098, respectively). The 0.1, 0.25, and 0.5 mg/mL groups likewise did not differ significantly from the ethanol vehicle after multiplicity adjustment (Holm-adjusted *p* ≥ 0.301). The untreated control did not differ significantly from the ethanol vehicle (OR = 1.15, 95% CI 0.56–2.34, Holm-adjusted *p* = 0.709).

At 24 h, none of the caffeic acid concentrations differed significantly from the ethanol vehicle after Holm adjustment (all adjusted *p* ≥ 0.792). The untreated control also did not differ significantly from the ethanol vehicle (OR = 0.86, 95% CI 0.42–1.77, Holm-adjusted *p* = 1.000).

Comparisons between the independent 4 and 24 h cohorts identified a significant reduction in melanization odds at 24 h only in the 2.5 mg/mL group (OR = 0.14, 95% CI 0.03–0.56, Holm-adjusted *p* = 0.043). No significant between-cohort differences were detected for the untreated control, ethanol vehicle, 0.1, 0.25, 0.5, 1, or 5 mg/mL groups after multiplicity adjustment (Holm-adjusted *p* ≥ 0.128). These comparisons represent differences between independently sampled larval cohorts rather than longitudinal changes within the same individuals.

### 3.4. Effect of Caffeic Acid on Total Hemolymph Protein (TP) Concentration

Descriptive statistics, assumption checks, omnibus tests, and planned vehicle contrasts for total protein, SOD, CAT, and MDA are provided in Supplementary File S3 (Tables S12–S15).

**Figure 2:**
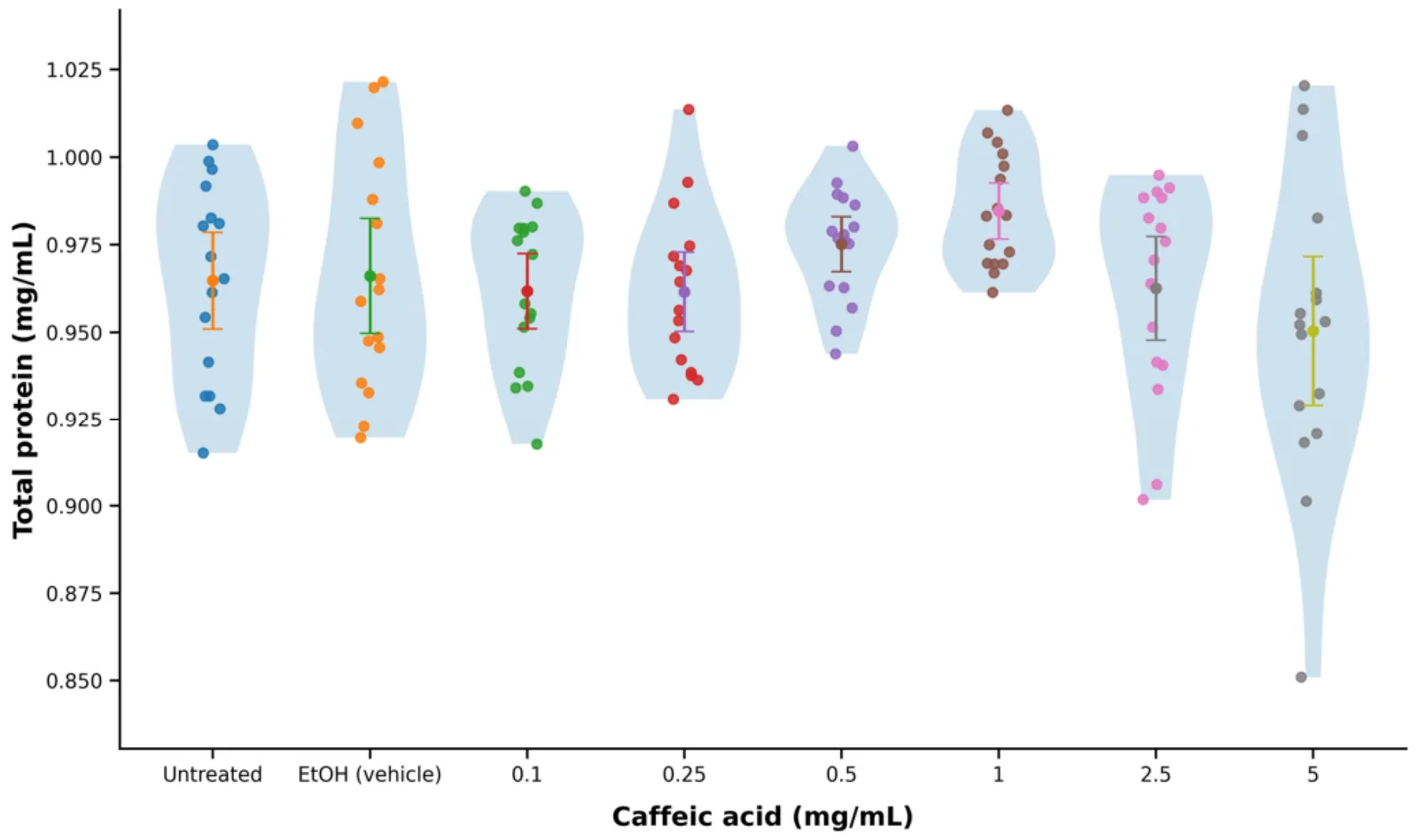
Effect of caffeic acid on total hemolymph protein concentration in *G. mellonella* larvae 24 h after treatment. Data are presented as mean ± SD (*n* = 16 larvae per group). Total protein was analyzed using an HC3-robust linear model including treatment and experimental run as fixed effects. After adjustment for experimental run, the treatment effect was significant (F(7, 117) = 3.28, *p* = 0.003, partial η^2^ = 0.164), and the experimental run effect was also significant (F(3, 117) = 5.83, *p* < 0.001). Planned comparisons against the ethanol vehicle were adjusted using the Holm procedure; no individual caffeic acid concentration differed significantly from the vehicle. Effect estimates and 95% CIs for all planned vehicle contrasts are provided in Supplementary File S3 (Tables S14 and S15).

Total hemolymph protein concentrations showed modest variation among the experimental groups (Figure 2). Because the assumption of homogeneity of variances was not met, TP was analyzed using an HC3-robust linear model including treatment and experimental run as fixed effects, with run serving as a blocking factor. After adjustment for experimental run, the overall treatment effect remained significant (F(7, 117) = 3.28, *p* = 0.003, partial η^2^ = 0.164), and the experimental run effect was also significant (F(3, 117) = 5.83, *p* < 0.001; Supplementary File S3, Table S14). Mean protein concentrations ranged from 0.950 ± 0.043 mg/mL in the 5 mg/mL group to 0.985 ± 0.016 mg/mL in the 1 mg/mL group.

Because caffeic acid was administered in 20% ethanol, the ethanol vehicle was used as the primary comparator. Run-adjusted planned comparisons against the vehicle, with Holm adjustment for multiple testing, revealed no significant differences for any caffeic acid concentration. Effect estimates and their 95% CIs for all planned vehicle contrasts are provided in Table S15. Thus, although the run-adjusted model indicated overall treatment heterogeneity, no individual caffeic acid concentration produced a statistically significant change in total hemolymph protein relative to the vehicle.

### 3.5. Effect of Caffeic Acid on Catalase (CAT) Activity

**Figure 3:**
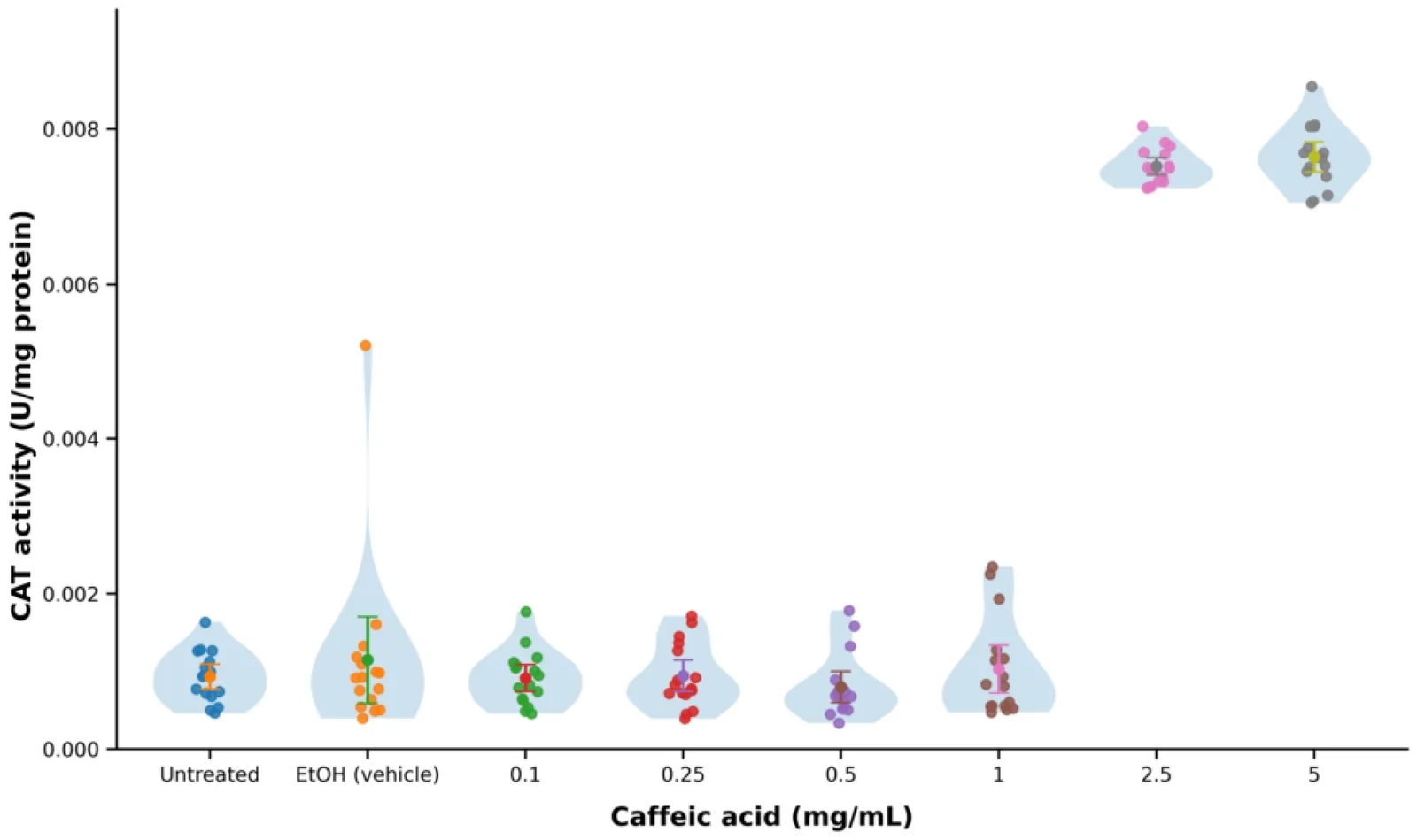
Effect of caffeic acid on catalase (CAT) activity in *G. mellonella* larvae 24 h after treatment. Because CAT activity was non-normally distributed, data are presented as median and interquartile range (*n* = 16 larvae per group). The Kruskal–Wallis analysis indicated a significant overall treatment effect (H(7) = 73.15, *p* < 0.001, ε^2^H = 0.551). In the run-adjusted rank-based fixed-block model, the treatment effect remained significant (HC3-robust F(7, 117) = 47.55, *p* < 0.001, rank-based partial η^2^ = 0.740), whereas the experimental run effect was not significant (F(3, 117) = 1.11, *p* = 0.350). Run-adjusted planned rank contrasts against the ethanol vehicle, with Holm adjustment for multiple testing, showed significantly higher CAT activity at 2.5 and 5 mg/mL caffeic acid (both Holm-adjusted *p* < 0.001). Corresponding effect estimates and 95% CIs are provided in Supplementary File S3 (Tables S14 and S15).

CAT activity showed marked heterogeneity among the experimental groups (Figure 3). Because CAT activity deviated from normality in several treatment groups, differences among groups were evaluated using the Kruskal–Wallis test. The analysis revealed a significant overall treatment effect (Kruskal–Wallis H(7) = 73.15, *p* < 0.001, ε^2^H = 0.551). In the run-adjusted rank-based fixed-block model, the treatment effect likewise remained significant (HC3-robust F(7, 117) = 47.55, *p* < 0.001, rank-based partial η^2^ = 0.740), whereas the experimental run effect was not significant (F(3, 117) = 1.11, *p* = 0.350; Supplementary File S3, Table S14).

Because of the non-normal distribution, CAT activity was summarized using medians and interquartile ranges. Median CAT activity was 0.000919 U/mg protein in the ethanol vehicle group, compared with 0.000885, 0.000799, 0.000685, 0.000818, 0.007497, and 0.007633 U/mg protein in the 0.1, 0.25, 0.5, 1, 2.5, and 5 mg/mL groups, respectively. The 2.5 and 5 mg/mL groups therefore showed substantially higher CAT activity than the lower-concentration and control groups.

To determine whether the overall heterogeneity represented caffeic-acid-associated differences relative to the vehicle, run-adjusted planned rank contrasts were performed using the ethanol vehicle as the reference, with Holm adjustment for multiple comparisons. CAT activity was significantly higher at both 2.5 and 5 mg/mL than in the ethanol vehicle group (both Holm-adjusted *p* < 0.001). The corresponding adjusted rank differences were 60.19 (95% CI 46.64–73.73) and 63.31 (95% CI 49.84–76.79), respectively (Supplementary File S3, Table S15). None of the remaining caffeic acid concentrations differed significantly from the vehicle after adjustment. Thus, the complete *n* = 16 dataset identified a concentration-specific increase in CAT activity at the two highest caffeic acid concentrations.

### 3.6. Effect of Caffeic Acid on Superoxide Dismutase (SOD) Activity

**Figure 4:**
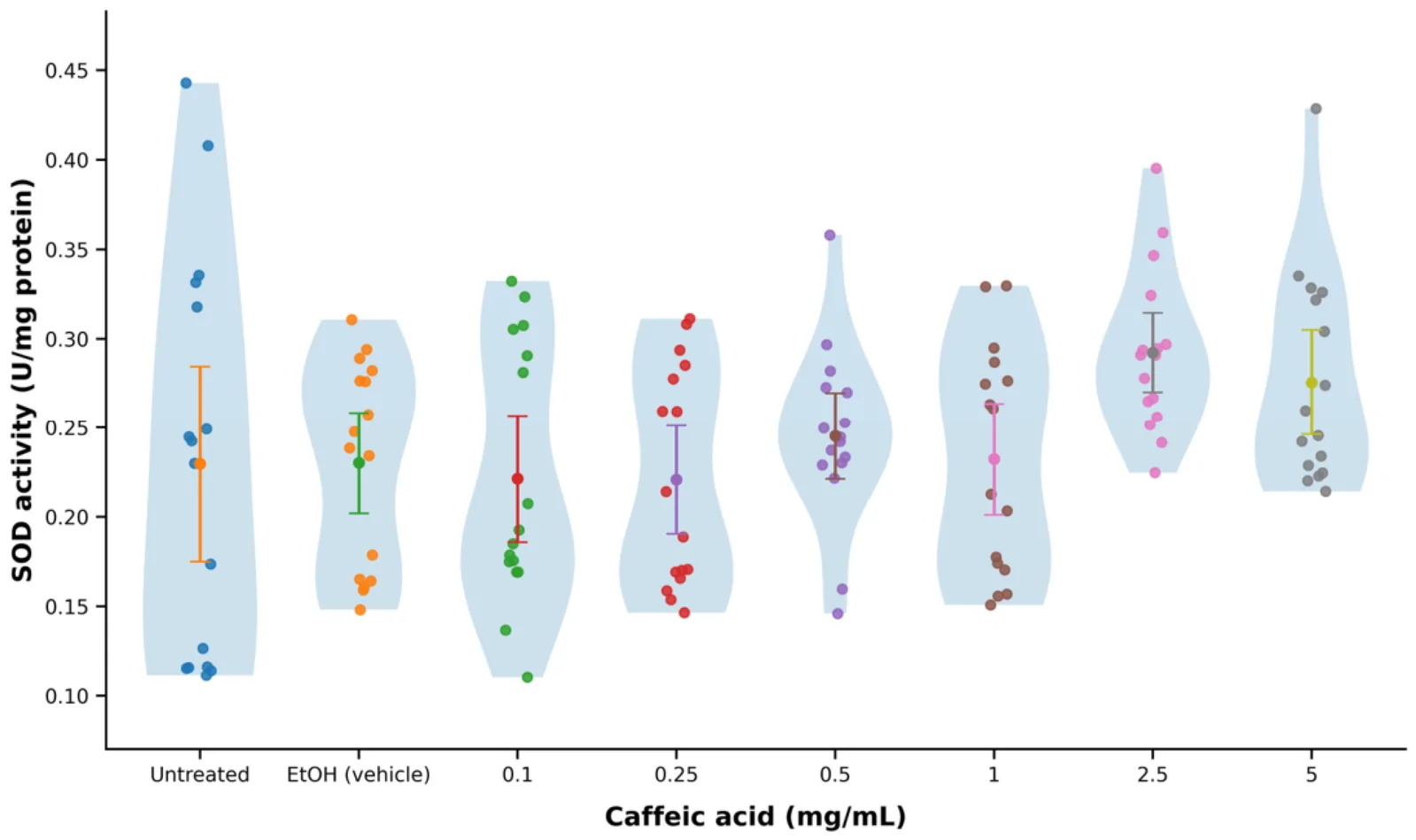
Effect of caffeic acid on superoxide dismutase (SOD) activity in *G. mellonella* larvae 24 h after treatment. Data are presented as mean ± SD (*n* = 16 larvae per group). SOD activity was analyzed using an HC3-robust linear model including treatment and experimental run as fixed effects. After adjustment for experimental run, the treatment effect was significant (F(7, 117) = 3.88, *p* < 0.001, partial η^2^ = 0.188), and the experimental run effect was also significant (F(3, 117) = 34.99, *p* < 0.001). Planned comparisons against the ethanol vehicle were adjusted using the Holm procedure. SOD activity was significantly higher only at 2.5 mg/mL caffeic acid (adjusted mean difference = 0.062 U/mg protein, 95% CI 0.030–0.094 U/mg protein, Holm-adjusted *p* = 0.0016). Detailed effect estimates and 95% CIs are provided in Supplementary File S3 (Tables S14 and S15).

SOD activity showed significant heterogeneity among the experimental groups (Figure 4). Because variance homogeneity and normality were not adequately satisfied across all groups, SOD activity was analyzed using an HC3-robust linear model including treatment and experimental run as fixed effects. After adjustment for experimental run, the treatment effect remained significant (F(7, 117) = 3.88, *p* < 0.001, partial η^2^ = 0.188), and a significant experimental run effect was also detected (F(3, 117) = 34.99, *p* < 0.001; Supplementary File S3, Table S14).

Mean SOD activity was 0.230 ± 0.058 U/mg protein in the ethanol vehicle group. Mean values in the caffeic acid-treated groups were 0.221 ± 0.073, 0.221 ± 0.062, 0.245 ± 0.049, 0.232 ± 0.064, 0.292 ± 0.045, and 0.276 ± 0.060 U/mg protein at 0.1, 0.25, 0.5, 1, 2.5, and 5 mg/mL, respectively. The highest mean SOD activity was observed at 2.5 mg/mL.

Run-adjusted planned comparisons against the ethanol vehicle, with Holm adjustment for multiple testing, showed that SOD activity was significantly higher only at 2.5 mg/mL (adjusted mean difference = 0.062 U/mg protein, 95% CI 0.030–0.094 U/mg protein, Holm-adjusted *p* = 0.0016; Supplementary File S3, Table S15). The 5 mg/mL group showed a numerical increase relative to the vehicle, but this difference did not remain significant after multiplicity adjustment (Holm-adjusted *p* = 0.062). None of the other caffeic acid concentrations differed significantly from the ethanol vehicle. Effect estimates and 95% CIs for all planned vehicle contrasts are provided in Supplementary File S3, Table S15. These results therefore indicate a selective increase in SOD activity at 2.5 mg/mL rather than a monotonic concentration-dependent response.

### 3.7. Effect of Caffeic Acid on Malondialdehyde (MDA) Levels

**Figure 5:**
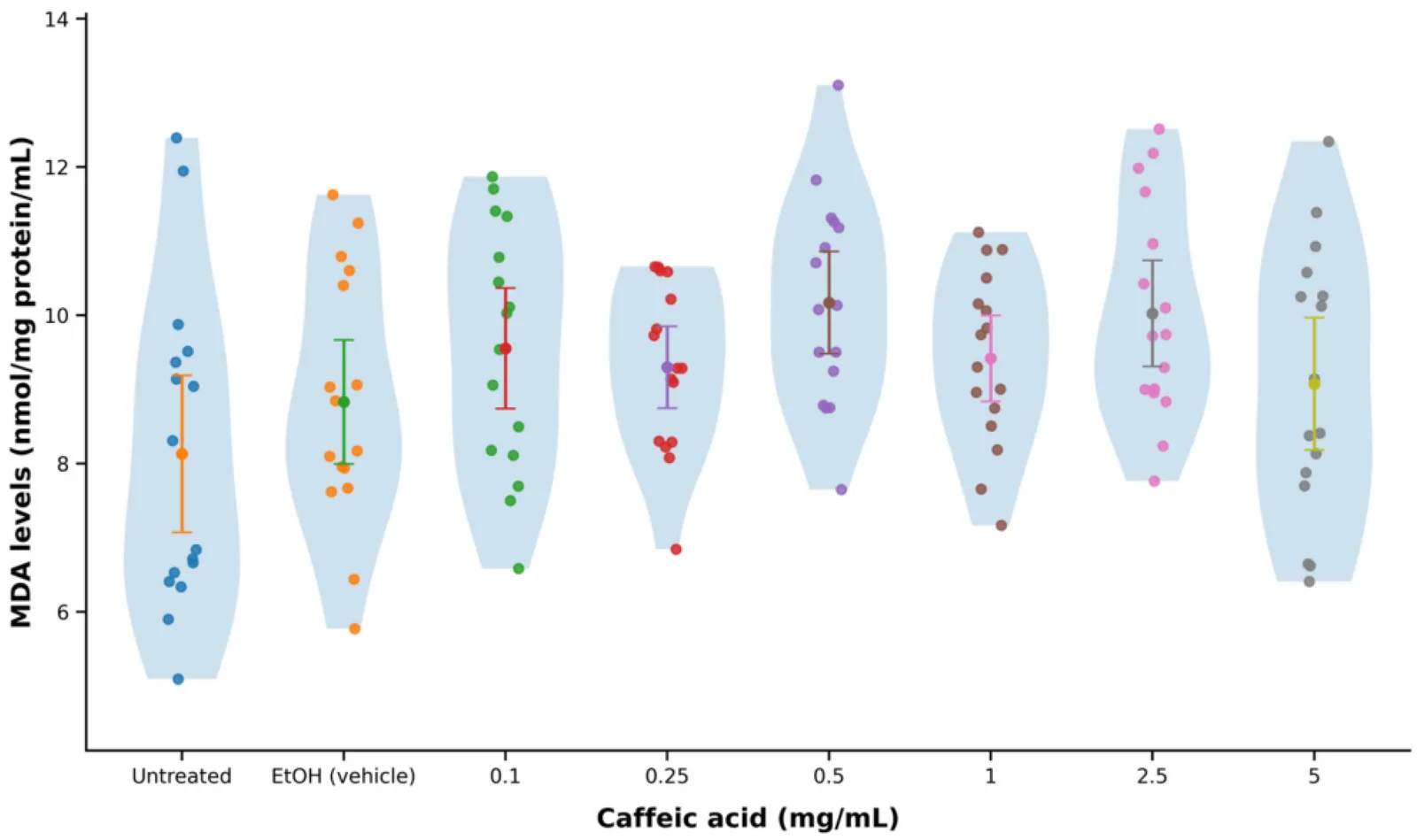
Effect of caffeic acid on malondialdehyde (MDA) levels in *G. mellonella* larvae 24 h after treatment. Data are presented as mean ± SD (*n* = 16 larvae per group). MDA was analyzed using a linear model including treatment and experimental run as fixed effects, with run serving as a blocking factor. After adjustment for experimental run, the treatment effect was significant (F(7, 117) = 3.10, *p* = 0.005, partial η^2^ = 0.156), and the experimental run effect was also significant (F(3, 117) = 7.14, *p* < 0.001). Planned comparisons against the ethanol vehicle were adjusted using the Holm procedure; no individual caffeic acid concentration differed significantly from the vehicle after adjustment. Effect estimates and 95% CIs for all planned vehicle contrasts are provided in Supplementary File S3 (Tables S14 and S15).

MDA levels satisfied the assumptions required for parametric analysis and were therefore evaluated using a linear model including treatment and experimental run as fixed effects, with run serving as a blocking factor (Figure 5). After adjustment for experimental run, the overall treatment effect remained significant (F(7, 117) = 3.10, *p* = 0.005, partial η^2^ = 0.156), and the experimental run effect was also significant (F(3, 117) = 7.14, *p* < 0.001; Supplementary File S3, Table S14).

Mean MDA levels were 8.83 ± 1.71 nmol/mg protein/mL in the ethanol vehicle group and 9.55 ± 1.66, 9.30 ± 1.13, 10.17 ± 1.41, 9.42 ± 1.18, 10.02 ± 1.46, and 9.07 ± 1.82 nmol/mg protein/mL in the 0.1, 0.25, 0.5, 1, 2.5, and 5 mg/mL groups, respectively. The highest mean MDA level was observed at 0.5 mg/mL, followed by 2.5 mg/mL.

Because the ethanol vehicle was the primary comparator, run-adjusted planned comparisons were performed against the vehicle with Holm adjustment for multiple testing. None of the caffeic acid concentrations differed significantly from the ethanol vehicle after adjustment (minimum Holm-adjusted *p* = 0.086). Effect estimates and 95% CIs for all planned vehicle contrasts are provided in Supplementary File S3, Table S15. Although numerical increases were observed at some concentrations, particularly 0.5 and 2.5 mg/mL, these differences were not statistically supported after multiplicity correction. Therefore, the significant overall treatment effect indicates heterogeneity among groups but does not support attribution of altered MDA levels to any specific caffeic acid concentration relative to the ethanol vehicle.

## 4. Discussion

The present study demonstrates that caffeic acid produces non-uniform and concentration-specific effects on the physiology of *G. mellonella*, with temporal variation being particularly evident in the encapsulation response. The clearest responses were observed in cellular and bead-associated immune endpoints, together with concentration-specific changes in antioxidant enzyme activities at the higher caffeic acid concentrations. Total hemocyte count (THC) showed a non-monotonic response, with a significant increase relative to the ethanol vehicle only at 0.5 mg/mL. Encapsulation exhibited a pronounced treatment × time interaction, whereas melanization remained high across all groups and showed only limited concentration-specific differences. At 4 h, melanization was significantly greater than the ethanol vehicle only at 2.5 mg/mL after multiplicity adjustment, while no caffeic acid concentration differed significantly from the vehicle at 24 h. Among the biochemical endpoints measured at 24 h, CAT activity was significantly increased at 2.5 and 5 mg/mL and SOD activity was significantly increased at 2.5 mg/mL relative to the ethanol vehicle. MDA levels and total hemolymph protein showed significant overall heterogeneity among experimental groups, but no individual caffeic acid concentration differed significantly from the vehicle after multiplicity adjustment. Collectively, these findings indicate that caffeic acid influences immune-associated and redox-related physiology in an endpoint-specific manner rather than producing a uniform concentration-dependent response.

Total hemocyte count provides an indicator of circulating cellular immune status, but changes in THC can arise through several processes, including mobilization between sessile and circulating hemocyte populations, tissue recruitment, adhesion, survival, proliferation, and hematopoietic activity. Insect hemocytes also perform functions extending beyond classical cellular immunity, emphasizing that variation in circulating hemocyte abundance cannot be attributed to a single physiological mechanism [26]. In *G. mellonella*, plasmatocytes and granulocytes constitute major adhesive and phagocytic hemocyte populations, and circulating hemocyte abundance may change rapidly following immune or physiological challenge [27,28,29].

In the present study, THC exhibited a non-monotonic response. The 0.5 mg/mL group showed the highest mean THC and was the only caffeic acid concentration that remained significantly different from the ethanol vehicle after multiplicity adjustment. Increasing the caffeic acid concentration beyond 0.5 mg/mL did not produce a further increase in circulating hemocytes, with mean THC instead returning toward or below vehicle-associated levels. The response therefore cannot be described as a progressive concentration-dependent increase in hemocyte abundance. Rather, the data indicate a concentration-specific peak within the intermediate exposure range. A more densely resolved concentration series would be required to determine the precise form of this response.

Evidence from other insect and mammalian systems supports the broader possibility that caffeic acid can influence cellular immune physiology, although direct mechanistic comparisons should be made cautiously. Dietary caffeic acid has been reported to alter physiological and biochemical responses in *Spodoptera litura* in a concentration-dependent manner [30], while studies in human monocytes have demonstrated caffeic-acid-associated modulation of immune receptor expression, cytokine responses, and fungicidal activity [31]. These observations are consistent with the capacity of caffeic acid to modify immune-cell-related responses but do not establish that the same cellular mechanisms account for the THC pattern observed here. Moreover, chemical exposure studies in *G. mellonella* have shown that changes in total hemocyte abundance may occur independently of hemocyte subtype composition or other functional cellular immune endpoints [32]. Accordingly, the increased THC observed at 0.5 mg/mL should not be interpreted as direct evidence of enhanced hematopoiesis or generalized immunostimulation. Because sessile hemocytes, differential hemocyte populations, proliferation, viability, and apoptosis were not measured, the present result is most appropriately interpreted as an alteration in circulating hemocyte status.

The encapsulation response provided particularly clear evidence of treatment- and time-dependent modulation. Encapsulation represents a major cellular defense against foreign targets too large for phagocytosis and depends on coordinated recognition, adhesion, spreading, and cellular layering, particularly involving granulocytes and plasmatocytes [29,33]. Analysis of the ordered encapsulation categories demonstrated a pronounced treatment × time interaction. At 4 h, all caffeic acid concentrations increased the odds of assignment to a higher encapsulation category relative to the ethanol vehicle. At 24 h, however, the response diverged according to concentration: 0.1–1 mg/mL remained associated with greater odds of higher-category encapsulation, whereas 2.5 and 5 mg/mL shifted the response toward lower encapsulation categories. The complementary Encapsulation Severity Index supported this pattern and indicated that the high-concentration effect at 24 h primarily reflected redistribution from strong toward weak encapsulation rather than a generalized failure to encapsulate the beads.

This temporal divergence is biologically relevant because capsule formation is a dynamic process involving continued hemocyte recruitment, adhesion, spreading, and maturation. The present data therefore suggest that caffeic acid altered the distribution and persistence of encapsulation severity rather than exerting a simple uniformly stimulatory or suppressive effect. The stronger early response across caffeic acid treatments and the later shift toward weak encapsulation at 2.5 and 5 mg/mL may reflect differences in the kinetics or maintenance of capsule formation. However, because hemocyte recruitment, adhesion dynamics, subtype composition, and capsule maturation were not measured directly, these possibilities remain hypotheses rather than demonstrated mechanisms.

The ethanol vehicle also requires consideration in interpreting the encapsulation findings. The untreated control showed significantly greater odds of assignment to a higher encapsulation category than the ethanol vehicle at both assessment times. Thus, the vehicle itself influenced the encapsulation response, reinforcing the importance of using the ethanol-treated group rather than the untreated group as the primary comparator for caffeic-acid-associated effects. At the same time, the observation that several caffeic acid concentrations produced stronger encapsulation than the vehicle indicates that the treatment patterns cannot be explained solely by the presence of ethanol.

Melanization showed a substantially different response profile from encapsulation. In insects, melanization is closely associated with the prophenoloxidase system and the generation of reactive quinone intermediates that ultimately contribute to melanin formation and host defense [33]. In *G. mellonella*, melanization has been experimentally demonstrated to contribute directly to antifungal defense [34]. Because intermediates generated during melanization may also be damaging to host tissues, this response is subject to spatial and temporal regulation [33].

Visible bead melanization remained consistently high across all experimental groups at both assessment times. The updated bead-level analysis did not identify a significant overall treatment × time interaction, indicating that there was insufficient evidence that the treatment-associated pattern of melanization differed globally between the independently sampled 4 and 24 h cohorts. Nevertheless, planned contrasts revealed a limited concentration-specific effect at 4 h. Only the 2.5 mg/mL group showed significantly greater odds of melanization than the ethanol vehicle after Holm adjustment. Although melanization odds were numerically higher at 5 and 1 mg/mL, these differences did not remain significant after multiplicity correction, and the 0.1, 0.25, and 0.5 mg/mL groups also did not differ significantly from the vehicle. At 24 h, none of the caffeic acid concentrations differed significantly from the ethanol vehicle after adjustment for multiple comparisons.

The between-cohort analysis likewise provided limited evidence for temporal variation in melanization. Only the 2.5 mg/mL group showed significantly lower melanization odds in the 24 h cohort than in the corresponding 4 h cohort after Holm adjustment. No significant between-cohort difference was detected for the untreated control, ethanol vehicle, or the remaining caffeic acid concentrations. Because the 4 and 24 h measurements were obtained from independent larval cohorts, this difference should be interpreted as a between-cohort contrast rather than as longitudinal loss of melanization from individual beads or larvae. Overall, the melanization findings therefore indicate a relatively stable response with a localized early effect at 2.5 mg/mL rather than a broad concentration- or time-dependent shift.

Visible bead melanization should not be interpreted as a direct quantitative measurement of phenoloxidase activity. Pigmentation represents the integrated outcome of several processes, including proPO activation, substrate availability, quinone production, pigment polymerization, and the local cellular environment. Accordingly, the present results demonstrate differences in visible melanization but do not establish that caffeic acid directly increased or inhibited PO activity. Direct biochemical measurement of PO would be required to resolve this question.

Cellular immunity and melanization are closely linked to redox-active processes, but their relationship with systemic biochemical redox markers is not necessarily linear. Quinones and other reactive intermediates generated during melanization can contribute both to defense and oxidative burden, while ROS themselves participate in innate immune signaling and antimicrobial responses [35]. Previous work in *G. mellonella* has also demonstrated changes in enzymatic and non-enzymatic antioxidant defenses during encapsulation, supporting physiological interaction between cellular immune activity and maintenance of hemolymph redox balance [36]. In the present study, the simultaneous responses observed in encapsulation, selected melanization contrasts, SOD, and CAT indicate that both immune-associated and antioxidant-defense-related processes were responsive to caffeic acid exposure, although their concentration-response profiles were clearly not parallel.

Caffeic acid is well recognized for its radical-scavenging and metal-binding properties, but antioxidant activity demonstrated in chemical systems does not necessarily predict the response of an intact organism. In vivo effects depend on exposure conditions, concentration, metabolism, endogenous antioxidant capacity, and the prevailing redox environment [4]. Previous insect studies likewise demonstrate that phenolic or plant-derived compounds can produce endpoint-specific rather than uniform immune and oxidative responses. Dietary caffeic acid affected biochemical physiology in *S. litura* [30], and endpoint-specific immune and redox responses have recently been reported in *G. mellonella* exposed to Hypericum perforatum [37]. The present results are consistent with such endpoint specificity, particularly because the antioxidant enzymes did not exhibit identical concentration-response patterns.

CAT showed the most pronounced biochemical response. Activity differed substantially among experimental groups, and the 2.5 and 5 mg/mL caffeic acid groups showed significantly higher CAT activity than the ethanol vehicle after multiplicity adjustment. SOD activity also showed a significant overall treatment effect, but the vehicle-centered comparisons identified a significant increase only at 2.5 mg/mL. The overlap of increased SOD and CAT activity at 2.5 mg/mL is compatible with coordinated enhancement of enzymatic defenses involved in superoxide and hydrogen peroxide handling. At 5 mg/mL, however, CAT remained significantly elevated whereas the numerical increase in SOD did not remain significant after multiplicity correction. Thus, the enzyme responses were concentration-specific and should not be interpreted as a simple monotonic induction of the antioxidant system.

Importantly, increased SOD and CAT activities do not by themselves establish that caffeic acid exerted a net antioxidant effect. Increased antioxidant enzyme activity may reflect an adaptive response to altered redox conditions as well as enhanced antioxidant capacity. This distinction is particularly relevant to the MDA findings. MDA showed significant overall heterogeneity among the experimental groups, with the highest mean values observed at 0.5 and 2.5 mg/mL, but none of the individual caffeic acid concentrations differed significantly from the ethanol vehicle after Holm correction. Thus, the present data do not provide statistically supported evidence that caffeic acid either increased or decreased lipid peroxidation at a specific concentration relative to its vehicle. The combination of increased antioxidant enzyme activities at selected concentrations and the absence of significant vehicle-specific MDA differences therefore supports an interpretation of redox modulation rather than demonstrated antioxidant protection or pro-oxidant injury.

The biochemical pattern is also relevant to interpretation of the immune findings. At 2.5 mg/mL, increased SOD and CAT activities occurred alongside significantly greater melanization odds at 4 h and a later shift toward weaker encapsulation at 24 h. In contrast, the concentration associated with maximal THC, 0.5 mg/mL, did not show corresponding increases in antioxidant enzyme activities or a significant melanization difference relative to the vehicle. Thus, although some immune-associated and antioxidant responses converged at 2.5 mg/mL, the overall concentration-response profiles remained distinct across endpoints. These differences argue against a single coordinated concentration-response pathway linking circulating hemocyte abundance, encapsulation, melanization, and systemic antioxidant enzyme activity. Instead, caffeic acid appears to affect these endpoints with different concentration sensitivities and potentially different temporal dynamics. Because ROS production and redox-sensitive signaling were not measured directly, causal relationships among these responses cannot be established.

Total hemolymph protein showed significant overall heterogeneity among experimental groups, but none of the caffeic acid concentrations differed significantly from the ethanol vehicle after multiplicity adjustment. Thus, the overall test does not support attribution of the protein variation to a particular caffeic acid concentration. Total protein represents a heterogeneous pool of immune, metabolic, transport, and storage proteins, and proteomic studies in *G. mellonella* have demonstrated that individual immune-related proteins can exhibit distinct temporal abundance patterns that may not be reflected by changes in total protein concentration [38,39,40]. Consequently, the absence of a concentration-specific effect on total protein does not exclude regulation of individual proteins, but neither does the significant omnibus effect establish activation or suppression of a particular protein-mediated pathway.

Viewed together, the results reveal a complex response rather than a uniform stimulatory or inhibitory effect of caffeic acid. The intermediate concentration of 0.5 mg/mL was associated with the highest circulating hemocyte abundance, encapsulation showed pronounced treatment- and time-dependent redistribution across response categories, and melanization remained generally high with only a limited concentration-specific effect at 4 h. The biochemical endpoints provided additional evidence of concentration specificity: CAT was increased at 2.5 and 5 mg/mL, SOD was increased at 2.5 mg/mL, whereas MDA and total protein showed overall heterogeneity without significant individual caffeic acid–vehicle contrasts. These differing profiles indicate that immune-associated and redox-related endpoints should not be interpreted as components of a single monotonic concentration-response pathway.

Several limitations should be considered when interpreting these findings. First, caffeic acid was administered directly into the hemocoel, providing a controlled internal exposure but bypassing absorption, digestive metabolism, and other barriers relevant to dietary or environmental exposure. The administered doses should therefore be interpreted as experimental internal exposures rather than as environmentally or nutritionally equivalent doses. Second, the 20% ethanol vehicle itself influenced encapsulation, emphasizing the importance of the vehicle-controlled comparisons used in the analyses. Third, the study focused primarily on acute immune and biochemical responses and did not include dedicated measurements of mortality, body-mass change, pupation, developmental progression, or whole-body melanization. Consequently, the present data cannot determine whether responses observed at the higher concentrations were accompanied by generalized physiological stress or sublethal toxicity.

A further limitation concerns the temporal resolution of the biochemical analyses. SOD, CAT, MDA, and total protein were measured only at 24 h. Because redox responses can be transient, this single time point cannot determine whether the observed enzyme responses developed earlier, persisted throughout the exposure period, or represented a later compensatory response. Earlier changes in ROS formation, antioxidant activity, or lipid peroxidation may also have occurred and resolved before biochemical sampling. ROS production, PO activity, glutathione status, redox-sensitive signaling, differential hemocyte populations, viability, proliferation, and apoptosis were not measured, further limiting mechanistic interpretation. In addition, encapsulation and melanization were evaluated by a single investigator who was aware of treatment allocation; therefore, observer-related bias cannot be excluded for these microscopically scored endpoints.

Finally, caffeic acid contains a redox-active catechol moiety and may potentially interact chemically or optically with colorimetric or absorbance-based assays. Assay-specific caffeic-acid blanks, spike-recovery experiments, or interference controls were not performed for the Bradford, SOD, CAT, or MDA assays. This limitation is particularly important when interpreting the biochemical differences observed here, especially because CAT and SOD activities showed significant concentration-specific changes. Consequently, these enzyme responses should be interpreted as assay-derived biochemical differences that require confirmation using appropriate interference controls and, ideally, complementary redox measurements. Future studies incorporating assay-specific interference controls, multiple biochemical sampling times, direct ROS and PO measurements, differential hemocyte analysis, and independent indicators of toxicity would provide a stronger basis for defining the mechanisms and temporal dynamics of caffeic acid action.

Overall, the present study indicates that caffeic acid modifies both innate immune-associated and selected redox-related responses in *G. mellonella* in a concentration-specific and non-uniform manner, with temporal dependence being most clearly demonstrated for encapsulation. The immune response was characterized by an intermediate-concentration peak in THC, marked temporal variation in encapsulation, and a comparatively stable melanization response with a limited early increase at 2.5 mg/mL. Among the biochemical endpoints, CAT activity increased at 2.5 and 5 mg/mL and SOD activity increased at 2.5 mg/mL, whereas MDA and total protein did not show significant concentration-specific differences relative to the ethanol vehicle after multiplicity correction. These findings support a cautious interpretation in which caffeic acid alters selected components of cellular immunity and endogenous antioxidant defense without establishing a uniformly antioxidant, pro-oxidant, immunostimulatory, or immunosuppressive state.

## 5. Conclusions

This study demonstrates that caffeic acid produces non-uniform, concentration- and endpoint-specific modulation of immune-associated and redox-related physiology in *G. mellonella*, with temporal dependence most clearly evident in the encapsulation response. The distinct concentration-response patterns observed across circulating hemocytes, bead-associated immune responses, and antioxidant enzymes indicate that caffeic acid does not act as a uniformly immunostimulatory, immunosuppressive, antioxidant, or pro-oxidant agent under the present experimental conditions. Rather, its effects depend on both the physiological endpoint and exposure concentration. These findings provide an integrated in vivo characterization of free caffeic acid across cellular immune and redox-related endpoints in *G. mellonella* and establish a basis for future mechanistic studies incorporating direct redox measurements, toxicity endpoints, and assay-interference controls.

## Figures and Tables

**Table 1:** Odds ratios for assignment to higher encapsulation categories following caffeic acid treatment relative to the ethanol vehicle.

Caffeic Acid Concentration	4 h OR (95% CI)	Holm-Adjusted *p*	24 h OR (95% CI)	Holm-Adjusted *p*
0.1 mg/mL	2.21 (1.75–2.80)	<0.001	1.73 (1.34–2.25)	<0.001
0.25 mg/mL	3.17 (2.48–4.06)	<0.001	2.17 (1.62–2.92)	<0.001
0.5 mg/mL	3.19 (2.53–4.02)	<0.001	1.71 (1.27–2.29)	0.001
1 mg/mL	2.16 (1.76–2.64)	<0.001	1.70 (1.25–2.29)	0.001
2.5 mg/mL	1.90 (1.52–2.38)	<0.001	0.59 (0.44–0.78)	<0.001
5 mg/mL	3.04 (2.38–3.88)	<0.001	0.37 (0.27–0.49)	<0.001

Table note: Odds ratios (ORs) and 95% confidence intervals for assignment of beads to a higher encapsulation category (none < weak < strong) in caffeic acid-treated larvae relative to the ethanol vehicle at 4 and 24 h. Estimates were obtained from a cumulative-logit generalized estimating equation (GEE) with larva as the clustering unit and robust covariance estimation. OR > 1 indicates greater odds of assignment to a higher encapsulation category relative to the ethanol vehicle, whereas OR < 1 indicates greater tendency toward lower encapsulation categories. *p* values were adjusted for multiple comparisons within each time point using the Holm procedure.

**Table 2:** Effect of caffeic acid administration on the encapsulation response of *G. mellonella* larvae at 4 and 24 h after Sephadex A-25 bead injection.

Time	Treatment	ESI	None (%)	Weak (%)	Strong (%)
4 h	Untreated	1.580 ± 0.100	4.1 ± 5.1	33.8 ± 7.6	62.1 ± 7.3
	EtOH vehicle	1.378 ± 0.114	4.3 ± 3.9	53.7 ± 7.1	42.1 ± 8.7
	0.1 mg/mL	1.595 ± 0.096	4.7 ± 6.1	31.1 ± 5.5	64.2 ± 5.0
	0.25 mg/mL	1.686 ± 0.083	1.1 ± 2.0	29.2 ± 8.3	69.7 ± 8.0
	0.5 mg/mL	1.688 ± 0.072	0.5 ± 1.4	30.1 ± 5.7	69.3 ± 6.3
	1 mg/mL	1.588 ± 0.060	2.0 ± 2.8	37.2 ± 6.5	60.8 ± 5.5
	2.5 mg/mL	1.555 ± 0.081	2.7 ± 3.4	39.1 ± 5.1	58.2 ± 5.9
	5 mg/mL	1.670 ± 0.092	2.9 ± 3.2	27.1 ± 7.8	70.0 ± 7.9
24 h	Untreated	1.549 ± 0.165	4.8 ± 5.1	35.5 ± 12.3	59.7 ± 13.7
	EtOH vehicle	1.392 ± 0.153	4.3 ± 3.7	52.3 ± 11.3	43.4 ± 12.9
	0.1 mg/mL	1.556 ± 0.076	3.0 ± 3.9	38.5 ± 4.0	58.6 ± 4.7
	0.25 mg/mL	1.607 ± 0.107	5.8 ± 4.7	27.8 ± 6.8	66.5 ± 7.6
	0.5 mg/mL	1.540 ± 0.117	7.8 ± 6.1	30.4 ± 4.9	61.8 ± 6.6
	1 mg/mL	1.551 ± 0.113	2.3 ± 5.1	40.3 ± 7.2	57.4 ± 8.0
	2.5 mg/mL	1.248 ± 0.101	2.9 ± 4.1	69.4 ± 9.4	27.7 ± 8.8
	5 mg/mL	1.116 ± 0.101	2.5 ± 5.5	83.4 ± 7.5	14.1 ± 7.0

Table note: Values are mean ± SD (*n* = 16 larvae per treatment and time point). ESI was calculated as [(1 × weak) + (2 × strong)]/total recovered beads and ranges from 0 to 2. Category percentages are presented descriptively on the original scale. Primary inferential analysis of encapsulation was based on the ordinal cumulative-logit GEE presented in Table 1. Four- and 24 h observations originated from independent larval cohorts.

**Table 3:** Effect of caffeic acid administration on the melanization response of *G. mellonella* larvae 4 and 24 h after Sephadex A-25 bead injection.

Treatment	4 h Melanization (%)	24 h Melanization (%)
Untreated	95.88 ± 5.07	94.83 ± 4.93
Ethanol vehicle	95.72 ± 3.89	95.69 ± 4.41
0.1 mg/mL	94.25 ± 4.78	93.11 ± 5.72
0.25 mg/mL	97.42 ± 2.94	95.62 ± 3.63
0.5 mg/mL	97.67 ± 3.77	96.12 ± 4.10
1 mg/mL	98.62 ± 2.55	96.50 ± 4.63
2.5 mg/mL	99.49 ± 1.40	96.42 ± 4.20
5 mg/mL	98.51 ± 2.69	95.59 ± 5.24

Table Note: Values are presented as mean ± SD of the percentage of melanized beads per larva (*n* = 16 larvae per treatment at each time point). The 4 and 24 h observations were obtained from independent larval cohorts. Inferential analysis was performed using a binomial generalized estimating equation (GEE) with larva as the clustering unit, an exchangeable working correlation structure, and robust covariance estimation. The model included treatment, time, and their interaction. The treatment × time interaction was not statistically significant (Wald χ^2^_7_ = 11.26, *p* = 0.128). Planned treatment contrasts were performed against the ethanol vehicle within each assessment time, and between-cohort comparisons were performed between 4 and 24 h within each treatment, with *p* values adjusted using the Holm procedure within each contrast family.

## Data Availability

The data presented in this study are available within the article and in a public repository (Zenodo) at: https://doi.org/10.5281/zenodo.22223616 (accessed on 10 September 2026).

## Supplementary Materials

The following supporting information can be downloaded at: https://www.mdpi.com/article/10.3390/biomedicines14092116/s1, Table S1: Descriptive statistics for encapsulation severity index (ESI) and the percentages of unencapsulated, weakly encapsulated, and strongly encapsulated beads by treatment and assessment time; Table S2: Factorial tests for encapsulation outcomes, including treatment, time, and treatment × time effects; Table S3: Grouped-binomial sensitivity analysis of strong versus non-strong encapsulation; Table S4: Planned encapsulation contrasts against the ethanol vehicle within each assessment time; Table S5: Between-cohort time contrasts for encapsulation outcomes within each treatment; Table S6: Full pairwise comparisons for encapsulation outcomes; Table S7: Run-adjusted ordinal cumulative-logit GEE analysis of encapsulation category, including model diagnostics and planned treatment contrasts; Table S8: Larva-level descriptive statistics for melanization by treatment and assessment time; Table S9: Run-adjusted planned melanization contrasts against the ethanol vehicle within each assessment time; Table S10: Between-cohort melanization contrasts between 24 h and 4 h within each treatment; Table S11: Full run-adjusted binomial GEE model for melanization, including treatment × time interaction and experimental-run block tests; Table S12: Descriptive statistics for total protein (TP), superoxide dismutase (SOD), catalase (CAT), and malondialdehyde (MDA); Table S13: Assumption checks for TP, SOD, CAT, and MDA; Table S14: Run-adjusted primary model tests for treatment and experimental-run effects on TP, SOD, CAT, and MDA; Table S15: Run-adjusted planned contrasts of caffeic acid treatments against the ethanol vehicle for TP, SOD, CAT, and MDA; Table S16: Descriptive statistics for total hemocyte count (THC); Table S17: Assumption checks for THC, including group-wise Shapiro–Wilk tests, Levene’s test, and residual normality assessment; Table S18: Run-adjusted fixed-block linear model for THC; Table S19: Planned run-adjusted THC contrasts against the ethanol vehicle with Holm-adjusted p values and 95% confidence intervals.

## Abbreviations

The following abbreviations are used in this manuscript:

CAT	Catalase
MDA	Malondialdehyde
NBT	Nitro blue tetrazolium
PO	Phenoloxidase
proPO	Prophenoloxidase
PTU	N-phenylthiourea
ROS	Reactive oxygen species
SOD	Superoxide dismutase
TBA	Thiobarbituric acid
TCA	Trichloroacetic acid
THC	Total hemocyte count
